# Effects of Climate change on temperature and precipitation in the Lake Toba region, Indonesia, based on ERA5-land data with quantile mapping bias correction

**DOI:** 10.1038/s41598-023-29592-y

**Published:** 2023-02-13

**Authors:** Hendri Irwandi, Mohammad Syamsu Rosid, Terry Mart

**Affiliations:** 1 Physics Department, FMIPA Universitas Indonesia, Depok, 16424 Indonesia; 2 The National Research and Innovation Agency, Jl. M.H. Thamrin No. 8, Jakarta, 10340 Indonesia

**Keywords:** Climate sciences, Environmental sciences, Hydrology, Limnology

## Abstract

Climate change is a serious problem that can cause global variations in temperature and rainfall patterns. This global variation can affect the water availability of lakes. In this study, trends in temperature and rainfall in the Lake Toba area for 40 years (1981–2020) were analyzed using ERA5-Land data corrected with observation station data utilizing the quantile mapping bias correction method. Corrected ERA5-Land data were used in this study to show spatial patterns and trends. The Mann–Kendall and Sen slope tests were carried out to see the magnitude of the trend. A comparison of temperature and rainfall against their baseline period (1951–1980) was also investigated. The results of this study show that climate change has affected the trend of increasing temperature and rainfall in the Lake Toba area, with an increase in temperature of 0.006 °C per year and an average rainfall of 0.71 mm per year. In general, significant changes in the increase of temperature and rainfall occurred in the last decade, with an increase in temperature of 0.24 °C and rainfall of 22%. The study of the impact of climate change expected to be useful for policymakers in managing water resources in the Lake Toba area.

## Introduction

Current climate change poses a threat to people around the world^1^. Climate change has been characterised by an increase in the global average surface temperature of 1.09 °C observed during the period 1850–2020, and in the last decade, there has been a significant increase in the temperature of 0.95–1.2 °C. Another impact of climate change is the increased intensity and frequency of heavy rain events^2,3^. Rainfall is the most important factor^4,5^, as increased rainfall can exacerbate the impact of hydrometeorological disasters^6,7^. Therefore, the need to understand the impact of climate change is an important issue in the sustainable water resources management sector^8^.

Lake Toba is in the north of Sumatra Island, Indonesia. It is a national strategic area, which has great benefits for the tourism, agriculture, plantation and fishery sectors, with the industrial sector also being equally important^9–11^. Several studies have indicated the effects of climate change in the Lake Toba area, revealing increasing trends in temperature and decreasing trends in rainfall^12^. However, as previous studies only used data obtained from one climate station to represent the whole Lake Toba region, with time series data of less than 30 years, further research is needed to explain the impact of climate change on temperature and rainfall in the area^13^.

In recent years, the Global Climate Model (GCM) developed by climate centres around the world can produce higher resolutions that are generally more accurate^14,15^. As such, it can be used to study climate change both regionally and locally^16^. However, GCM outputs cannot be used to directly simulate climate variables at local or site-specific scales^17^. Several studies have shown that the original GCM still contains considerable bias as a result of the downscaling process or from systematic error results^18,19^. Accordingly, a bias correction approach was developed to overcome this problem^20–23^.

Bias correction is an approach based on a statistical transformation that attempts to adjust the distribution of the modelled data to be very similar to the observed climatic variables^24^. Currently, most bias correction methods aim to adjust the mean, variance and distribution of certain climate variables^25^. Several refractive correction methods have been developed, with quantile mapping (QM) being the most commonly used^26–28^. The QM method effectively eliminates model bias, not only for mean and interannual variability but also for extreme events^24,29–31^. The QM bias correction method has also been developed and applied to the climate change impact studies in Italy^32^ and Sweden^30^ and was used to fill in missing climate data^33^.

To implement various adaptation and mitigation programmes in the future, impact assessment and climate change projections in an area are needed^34^. Detection and quantification of trends in climate variables is an important step for impact assessment and climate change projections^35–37^. Trend analysis can be carried out using the parametric method with the assumption that the data are normal, while for independent data sets that have outliers, it can be solved using non-parametric methods^38–42^. The Mann–Kendall (MK) trend test is used to determine the trend (positive or negative)^43,44^ while the Sen slope estimator (SSE)^45^ is used to determine the rate value of the trend in climate variables^46,47^.

The main objective of this study is to assess the impact of climate change on the trends in air temperature and rainfall in the Lake Toba region. The data used in this study, which are first corrected by the QM bias correction method, are high-resolution GCM data. It is believed that the results of this study will be vital for the government, society and policymakers in implementing adaptation, mitigation and water conservation programmes in the Lake Toba area.

## Methods

### Research location

**Figure 1:**
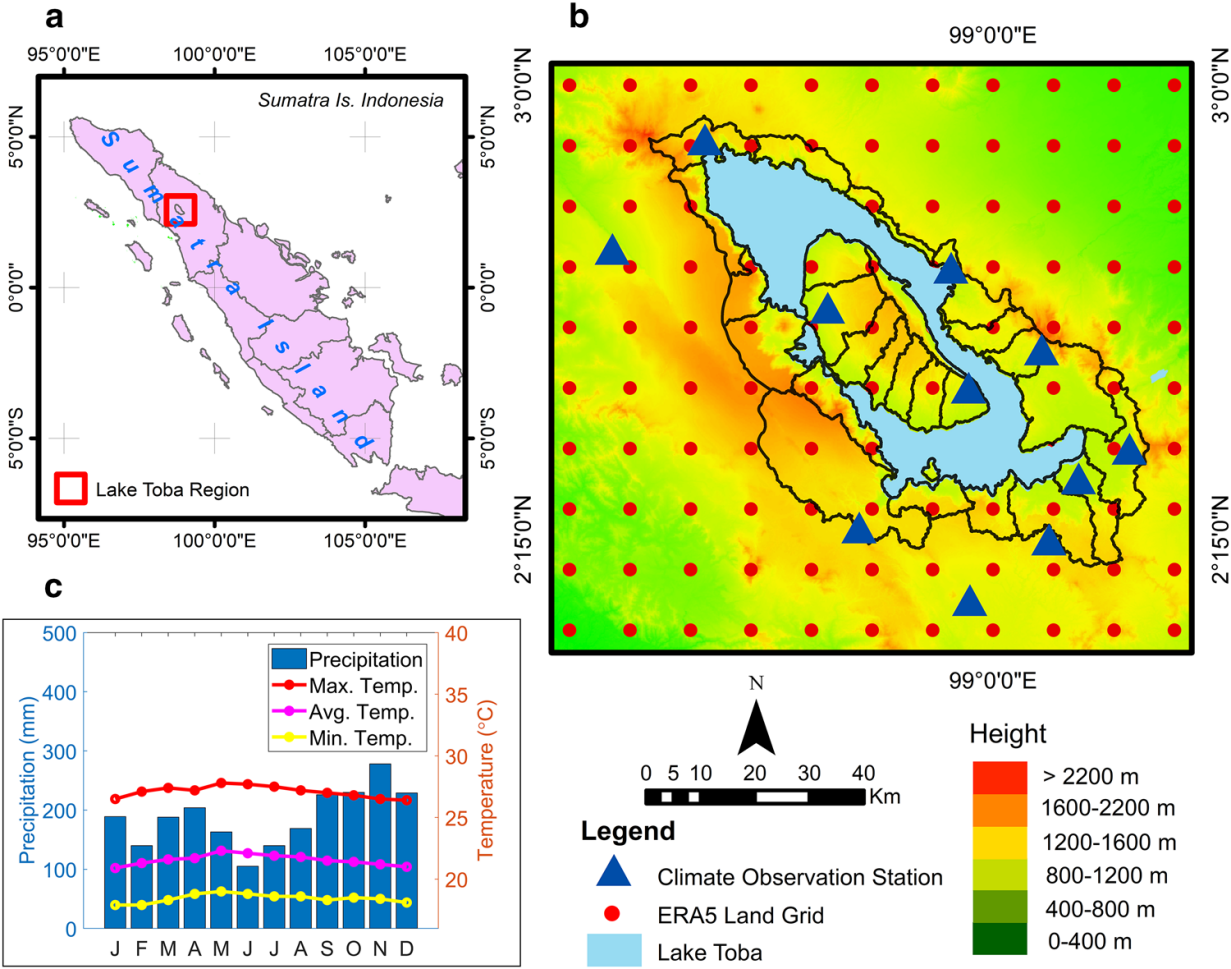
General description of the research area of the Lake Toba Region, (**a**) Located in the northern part of Sumatra Island, Indonesia, (**b**) Topography and Catchment Area of Lake Toba with Climate Observation Station, (**c**). Climagram of temperature and rainfall observation data.

Geographically, the Lake Toba area is located in the Bukit Barisan mountains in the northern part of Sumatra Island, Indonesia. The location is at 2.08–3.02° North Latitude and 98.28–99.32° East Longitude. The Lake Toba region also includes the catchment area of Lake Toba, with a water surface area of 1,124 km^2^ and a land area of 2,486 km^2^ in the catchment area (Fig. 1). The lake’s surface is at an altitude of 903 m above sea level, with a length of approximately 50 km, a width of about 27 km and an average depth of 228 m^48^. The Lake Toba area has a wet tropical climate with air temperatures ranging from 18 to 28 °C. The average minimum air temperature is 18.4 °C, the average maximum air temperature is 27.1 °C and the average air temperature is 21.5 °C^49^. Meanwhile, the annual rainfall is between 175 and 225 mm. The Lake Toba area has equatorial rain, with two rainy and dry seasons. The dry season takes place from December through February and June through August, while the rainy season spans from March through May and September through November^12^.

### Climate data

The climate observation data used in this study included rainfall data from 11 rainfall observation stations scattered in the Lake Toba area (Fig. 1). The data period used was from 1981 to 2020. The data were obtained from the Meteorology, Climatology and Geophysics Agency of the Deli Serdang Climatology Station. The distribution and length of climate observation data in the Lake Toba area were significantly constrained by the limited time series data. As such, the use of climate model data was also necessary. ERA5-Land data is a reanalysis dataset that provides a consistent view of the evolution of soil variables over decades with improved resolution compared to the ERA5 data. The reanalysis was performed by combining model data with observations from around the world to obtain a complete, globally consistent data set using the laws of physics. The spatial resolution of the ERA5-Land reanalysis data set was 9 km^50^.

### Quantile mapping bias correction

The QM method is a bias correction method that corrects the simulation model with the observation model by defining a transfer function: *y* = *f* (*x*). The transfer function relates the values of simulation and observation cumulative distribution functions (CDF)^29^ with the following equation:

1 $$ CDF_{{{\text{obs}}}} \left( {f\left( x \right)} \right) = CDF_{{{\text{sim}}}} \left( x \right) $$

where ${CDF}_{\mathrm{obs}}\left(f\left(x\right)\right)$ is the observation CDF and ${CDF}_{\mathrm{sim}}\left(x\right)$ is the simulation CDF.

To correct the bias using the quantitative mapping method, it is necessary to identify the distribution of the training data distribution. The data distribution is identified based on several parametric distribution Equations^51^. The distribution function that closely fits the data is selected based on the Akaike Information Criterion (AIC) value, while the used distributions include the normal (NOR), extreme value (EV), generalised extreme value (GEV), gamma (GAM), logistics (LOG), exponential (EXP), inverse Gaussian (ING), weibull (WB), log-normal (LN) and log-logistic (LL) values^52^. From the obtained distribution function, the quantile value of CDF will be used to construct the transfer function.

### Mean absolute error

The mean absolute error (MAE) is employed to validate the model that is used to correct the ERA5-Land data. MAE is expressed in the equation:

2 $$ MAE = \frac{1}{N}\mathop \sum \limits_{i = 1}^{n} \left| {x_{i} - y_{i} } \right| $$

where *x*_i_ is the rainfall observation data, *y*_i_ is the corrected ERA5-Land data and *N* is the amount of data.

### Mann–Kendall test

The identification of climate change was carried out using the nonparametric MK test^43,44^. This method is commonly used to identify the trends in time series of hydrometeorological data due to its insensitivity to the normal distribution of time series and data outliers. The MK test is recommended by the World Meteorological Organization as the best approach for assessing trends. This test can be summarised by the equation:

3 $$ S{ = }\mathop \sum \limits_{{k{ = 1}}}^{{n{ - 1}}} \mathop \sum \limits_{{j{ = }k{ + 1}}}^{n} {\text{Sgn}}\left( {{\text{x}}_{{\text{j}}} {\text{ - x}}_{{\text{k}}} } \right) $$

where *n* is the number of observations, *x*_j_ is the *j*th observation and Sgn (.) is the sign function, which represents

4 $$ {\text{Sgn}} \left( {x_{j} - x_{k} } \right) = \left\{ {\begin{array}{*{20}c} {1;} & {{\text{if}} x_{j} } & { > x_{i} } \\ {0;} & {{\text{if}} x_{j} } & { = x_{i} } \\ { - 1;} & {{\text{if}} x_{j} } & { < x_{i} } \\ \end{array} } \right\} $$

5 $$ {\text{Var }}\left( {\text{S}} \right){ = }\frac{{{\text{n}}\left( {\text{n - 1}} \right)\left( {\text{2n + 5}} \right) { - }\mathop \sum \nolimits_{{i{ = 1}}}^{m} {\text{t}}_{{\text{i}}} \left( {{\text{t}}_{{\text{i }}} { - 1}} \right)\left( {{\text{2t}}_{{\text{i}}} { + 5}} \right)}}{{{18}}} $$

After calculating the variance of *S* by using Eq. (5), the value of the standard test statistic (${\text{Z}}_{\text{mk}}$) can be calculated by using

6 $$ {\text{Z}}_{{\text{mk }}} { = }\left\{ {\begin{array}{*{20}c} {\frac{{\text{S - 1}}}{{\sqrt {{\text{Var}}\left( {\text{S}} \right)} }}{\text{ if S > 0}}} \\ {\text{ 0 if S = 0}} \\ {\frac{{\text{S + 1}}}{{\sqrt {{\text{Var}}\left( {\text{S}} \right)} }}{\text{ if S < 0}}} \\ \end{array} } \right. $$

A ${\text{Z}}_{\text{mk}}$ value higher (lower) than 1.96 (−1.96) indicates an increasing (decreasing) trend at the 95% confidence level (α = 0.05).

### Sen’s slope estimator test

Nonparametric methods were used to estimate the magnitude of trends in the time series data^45^. The slope of the data pair ‘n’ can be estimated in advance by using the following equation:

7 $$ \beta_{i} = {\text{median}}\left[ {\frac{{x_{j} - x_{k} }}{j - k}} \right]\forall \left( {k < j} \right), {\text{for}} i = 1,... n, $$

In Eq. (7), *x*_*j*_ and *x*_*k*_ represent the data values at times *j* and *k*, respectively, where *j* is always after *k* (*k* < *j*), βi is the slope estimator and *n* is the number of time periods. A negative *i* value represents a downward trend, while a positive *i* value represents an increasing trend over time.

## Results

This section presents the validation results of the QM bias correction model, where the corrected data will be used to generate the trends in the Lake Toba region. The trend analysis for climate change detection uses the MK statistical test (*Z*), Sen slope (*Q*) and significance level (SL). Positive and negative *Z* values indicate an up and down trend, respectively. The value of *Q* describes the magnitude or rate of trend change per year in a time series. The SL value shows how statistically significant or strong the trend signal is in the time series. Trend strength is divided into four categories based on the level of significance: (1) very weak trend (*α* > 0.1), (2) weak trend (0.05 < *α* < 0.1), (3) strong trend (0.01 < *α* < 0.05) and (4) very strong trend (*α* < 0.01), as shown in Figs. 3 and 4. Meanwhile, the impact period (1981–2020) is compared to the base period (1951–1980) to identify any changes, which are analysed per decade. The monthly and seasonal scale analyses presented in this study cover the period of the first dry season (December through February), the first rainy season (March through May), the second dry season (June through August) and the second rainy season (September through November).

### Quantile mapping bias correction

Table 1 shows the distribution of observed air temperature and ERA5-Land temperature, as well as observational rainfall and ERA5-Land rainfall. The observed temperature and ERA5-Land temperature tend to fit the generalised extreme value distribution approach, except in January, during which they are closer to the logistics distribution approach, while in April, October and December they tend to fit the Normal distribution approach. In the observation of rainfall, the tendency is to use the Weibull distribution, except in September and December, during which they tend to match the Gamma distribution approach and generalised extreme value. While in ERA5-Land rainfall, the tendency is to match the generalised extreme value distribution, in January they show the Log-normal distribution, in February the Inverse Gaussian distribution, in May and August the Gamma distribution and in June and September the generalised extreme value distribution. Moreover, October and December show the Log-logistics distribution. The differences in the results of distribution identification for each month are caused by the differences in the slope and symmetry of data distribution. Thus, a distribution pattern that can describe the observation data and ERA5-Land is needed.

**Table 1:** Identification of the data distribution of observation temperature and temperature of ERA5-Land and observation rainfall and rainfall of ERA5-Land.

No	Temperature	Jan	Feb	Mar	Apr	May	Jun	Jul	Aug	Sep	Oct	Nov	Dec
1	Observasi	LOG	GEV	GEV	NOR	GEV	GEV	GEV	GEV	GEV	NOR	GEV	NOR
2	ERA5-Land	GEV	GEV	GEV	GEV	GEV	GEV	GEV	GEV	GEV	GEV	GEV	GEV
**Rainfall**													
1	Observasi	WB	WB	WB	WB	WB	WB	WB	WB	GAM	WB	WB	GEV
2	ERA5-Land	LN	ING	GEV	GEV	GAM	WB	GEV	GAM	GEV	LL	GEV	LL

**Figure 2:**
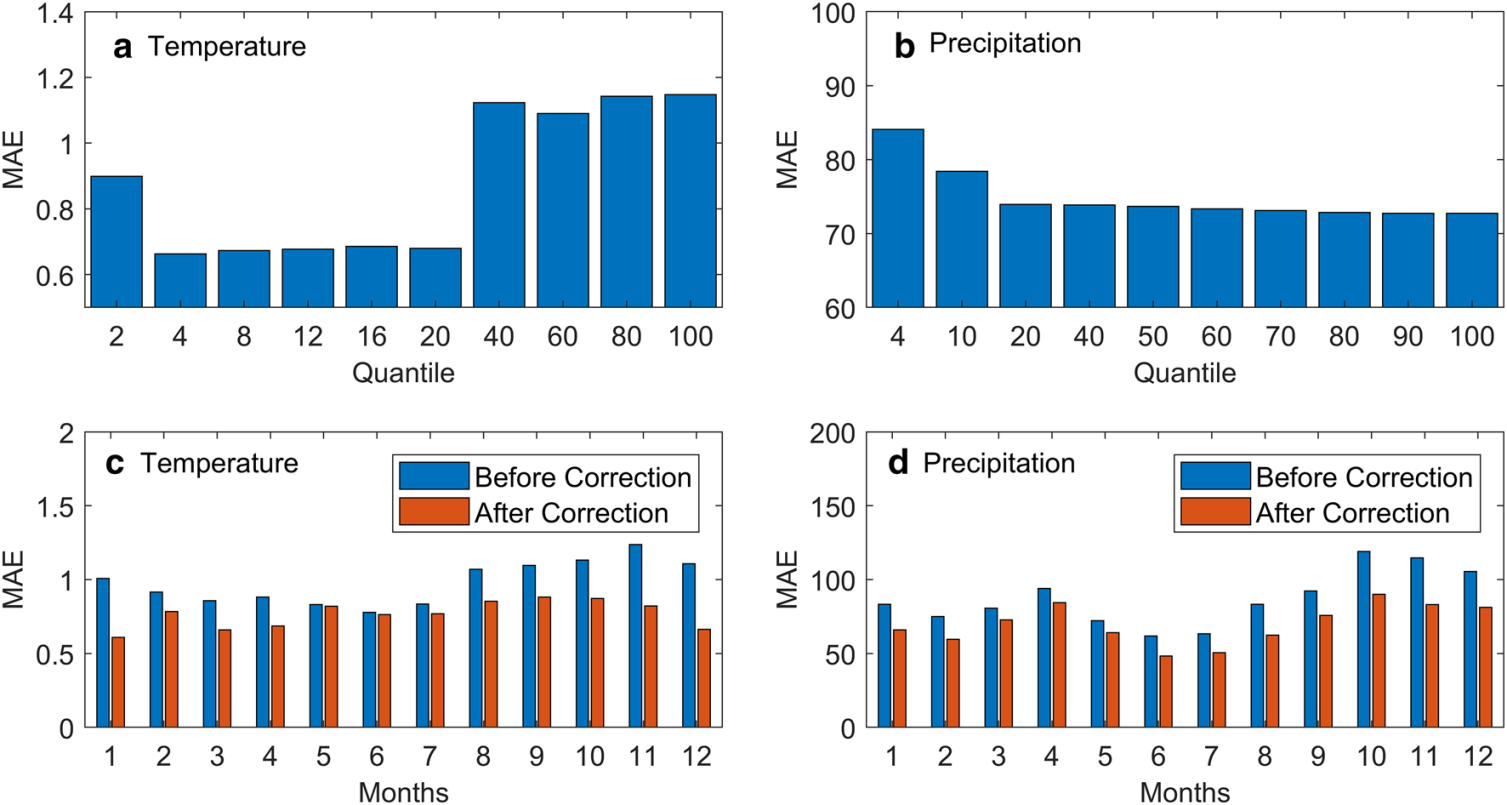
(**a**). The results of the validation of the temperature quantile value with *n* = 4, (**b**). The results of the validation of the rainfall quantile value with *n* = 80 and (**c**). Validation results for the temperature model and (**d**) for the rainfall model.

The accuracy of the model is determined by the quantile value. Therefore, it is necessary to validate the value by using the MAE value. The quantile values of 2, 4, 6, 8, 10, 14, 16, 18, 20, 40, 50, 60, 70, 80, 90 and 100 were tested to obtain the optimum MAE value. The most optimum MAE value for temperature was found to be at quantile 4 (Fig. 2a). As for rainfall, the optimum MAE value was observed at quantile 80, which is constant up to quantile 100 (Fig. 2b). In the case of temperature, the data do not show a large variation, so a small quantile value is sufficient. This differs from the rainfall that has a very high and heterogeneous variation. Consequently, a large quantile value is used to accommodate such conditions.

The MAE value determines the model’s accuracy so that the results of validation can verify whether this model is suitable or not. The validation of the corrected temperature model shows a better result compared to the model data that have not been corrected. As shown in Fig. 2c, the corrected MAE value is smaller than the uncorrected one. The same finding is also obtained in the case of rainfall validation (Fig. 2d).

### Temperature trend and temperature change

**Figure 3:**
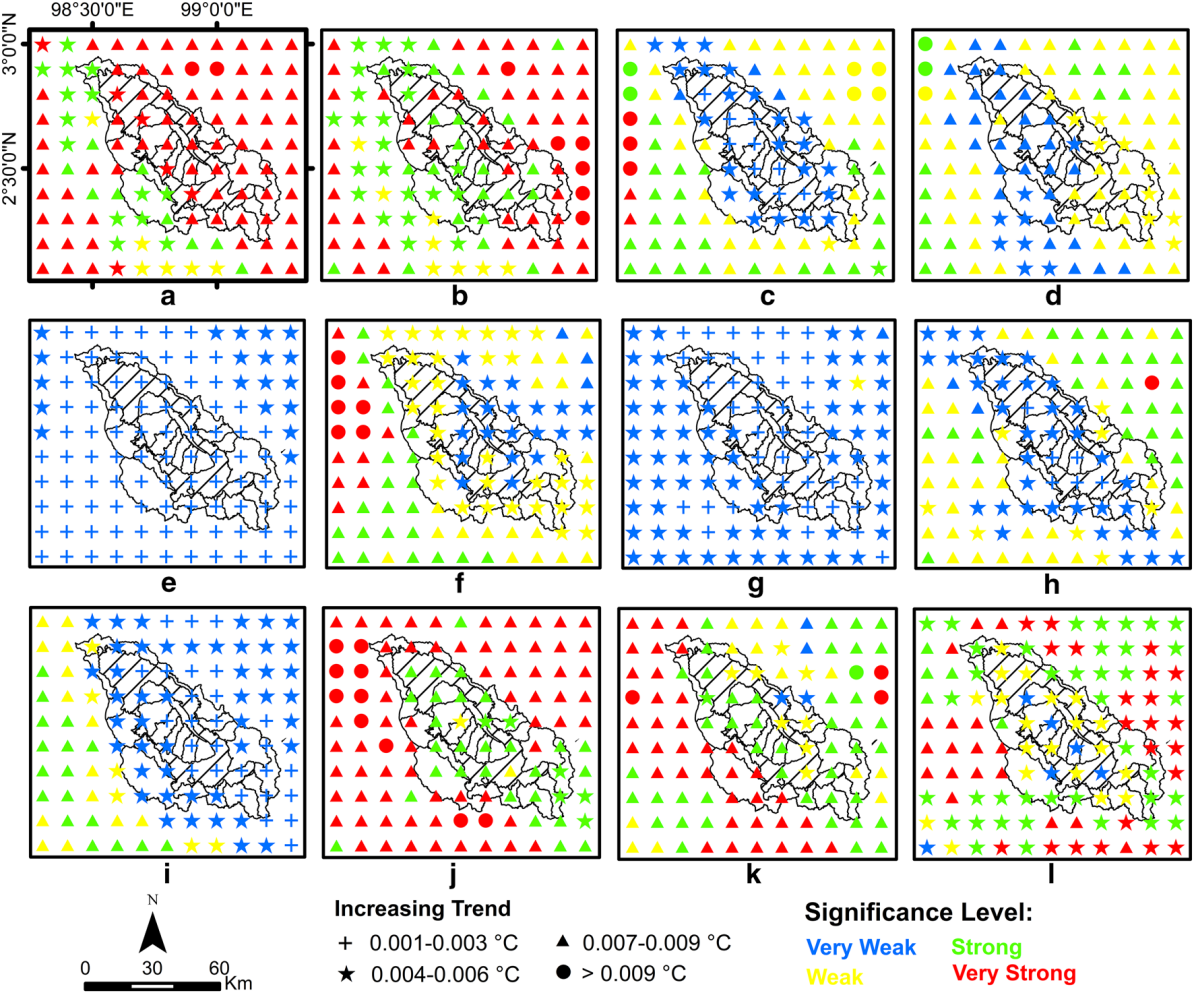
Spatial distribution of air temperature trends in the Lake Toba area for 1981–2020. In month (**a)** December, (**b**) January, (**c**) February, (**d**) March, (**e**) April, (**f**) May, (**g**) June, (**h**) July, (**i**) August, (**j**) September, (**k**) October, and (**l**) November.

Figure 3 shows the results of SL, *Z* and *Q*, the monthly temperatures in the Lake Toba region from 1981 to 2020. The spatial distribution of trends in the study area generally shows an increasing trend. An SL of 67% shows a strong to very strong increasing trend that occurred in December, January, March, May, September, October and November. In general, the trend of a weak temperature increase of 33% is spatially distributed in April and June. Meanwhile, in February, July and August, the trend of a weak temperature increase was generally distributed only around the Lake Toba catchment area. The rate of increasing temperature trends based on MK and *Q* shows a 50% rate of increase in temperature of 0.007–0.009 °C per year and a 33% rate of increase in temperature of 0.004–0.006 °C per year, while the rate of increase is 0.001–0.003 °C and > 0.009 °C per year, each by 13% and 3%, with an average increase rate of 0.006 °C per year. Generally, a strong trend of increasing temperature occurs in the first dry and rainy seasons, while the second dry seasons tend to have a weak increasing trend. Meanwhile, the rate of temperature increase in the Lake Toba catchment area did not rise significantly compared to outside of the Lake Toba catchment area, especially in the first and second dry seasons.

**Figure 4:**
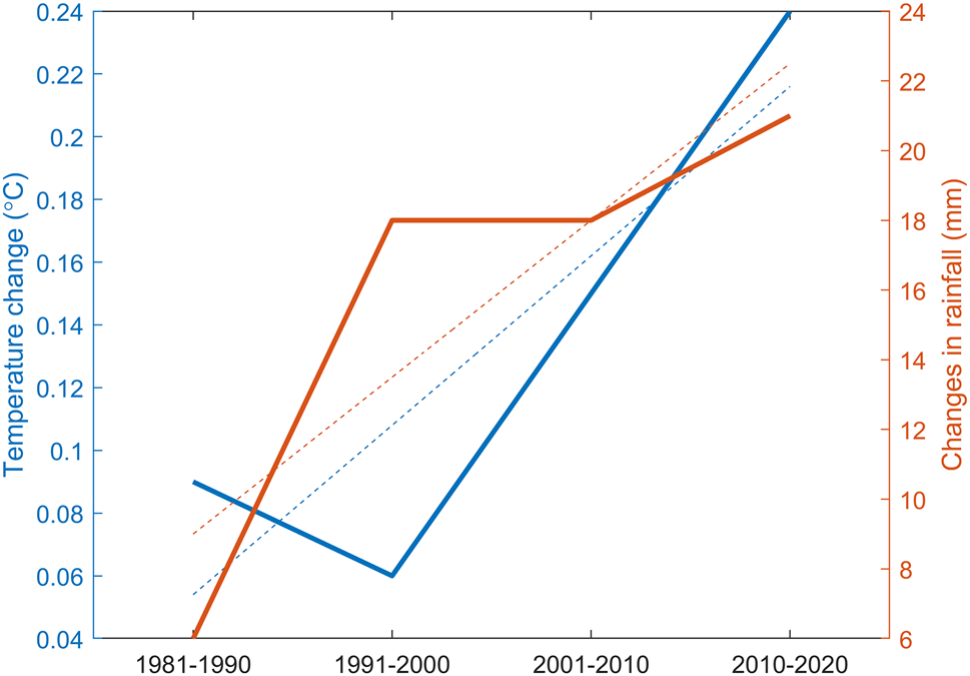
Changes in temperature (blue line) and rainfall (yellow line) relative to the base period (1951–1980).

**Figure 5:**
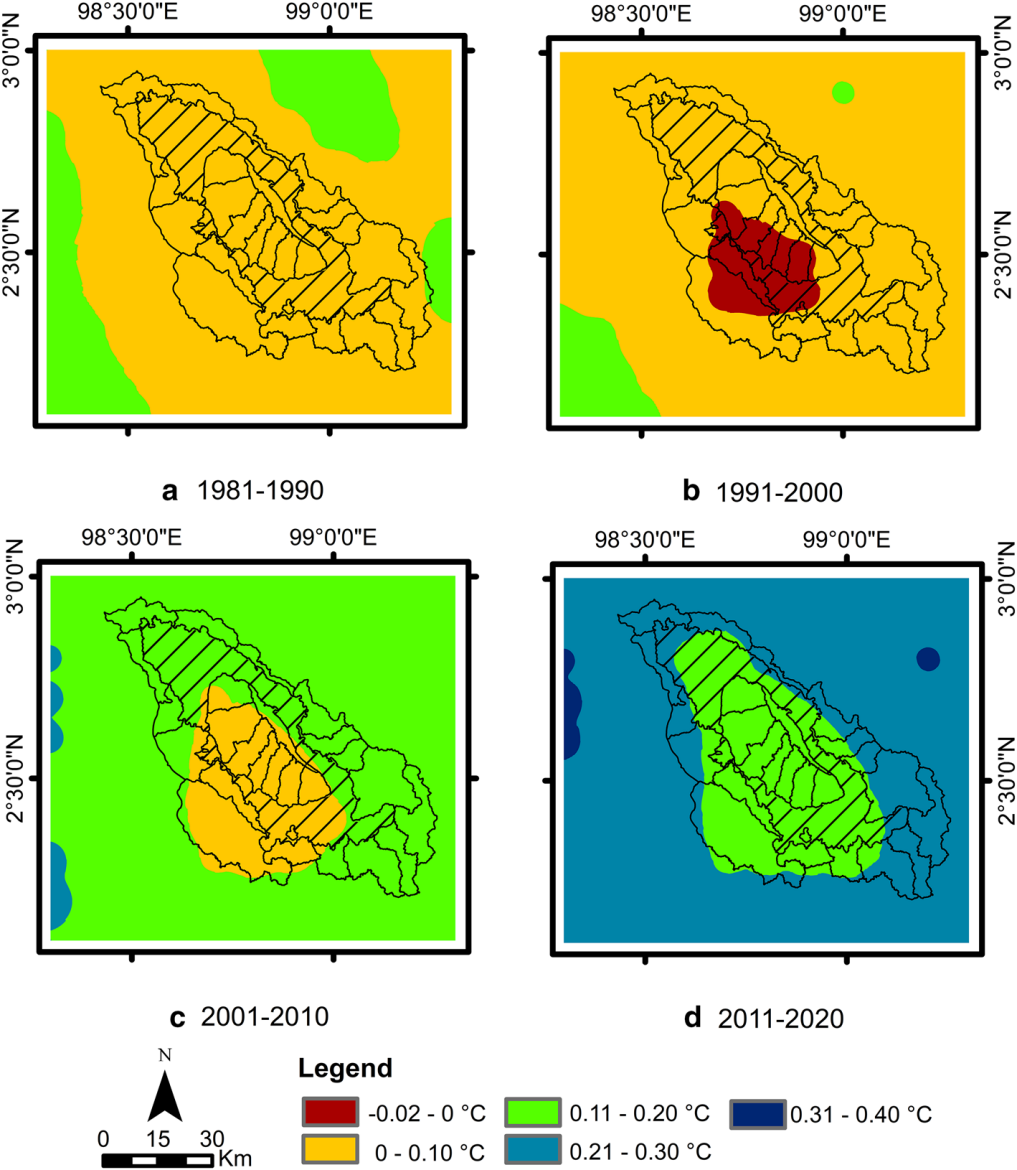
Spatial distribution of temperature changes in the decades (**a**) 1981–1990, (**b**) 1991–2000, (**c**) 2001–2010 and (**d**) 2011–2020 relative to the base period of 1951–1980.

Analyses of changes in temperature were carried out over the decades of 1981–1990, 1991–2000, 2001–2010 and 2011–2022, relative to the period of 1951–1980, to show the impact of temperature changes in the Lake Toba area (Fig. 4). In general, the temperature change, with an increasing trend every decade, is observed at the study site. The magnitudes of the temperature changes were 0.09 °C, 0.06 °C, 0.15 °C and 0.24 °C, respectively. The spatial distribution shown in Fig. 5 also indicates a similar tendency, that is, in the first two decades there was an increase of 0.1–0.2 °C, except that the southern part of the Lake Toba catchment area showed a temperature decrease of about 0.002 °C in the second decade. In the third decade, the temperature generally increases by 0.11–0.2 °C, except in the southern part of the Lake Toba catchment area, where there is an increase of 0.1 °C. While the last decade was the hottest, with a significant increase in temperature ranging from 0.21 to 0.3 °C, in some areas of Lake Toba, there was a lower temperature increase (i.e. 0.11–0.2 °C).

### Precipitation trends and changes in precipitation

**Figure 6:**
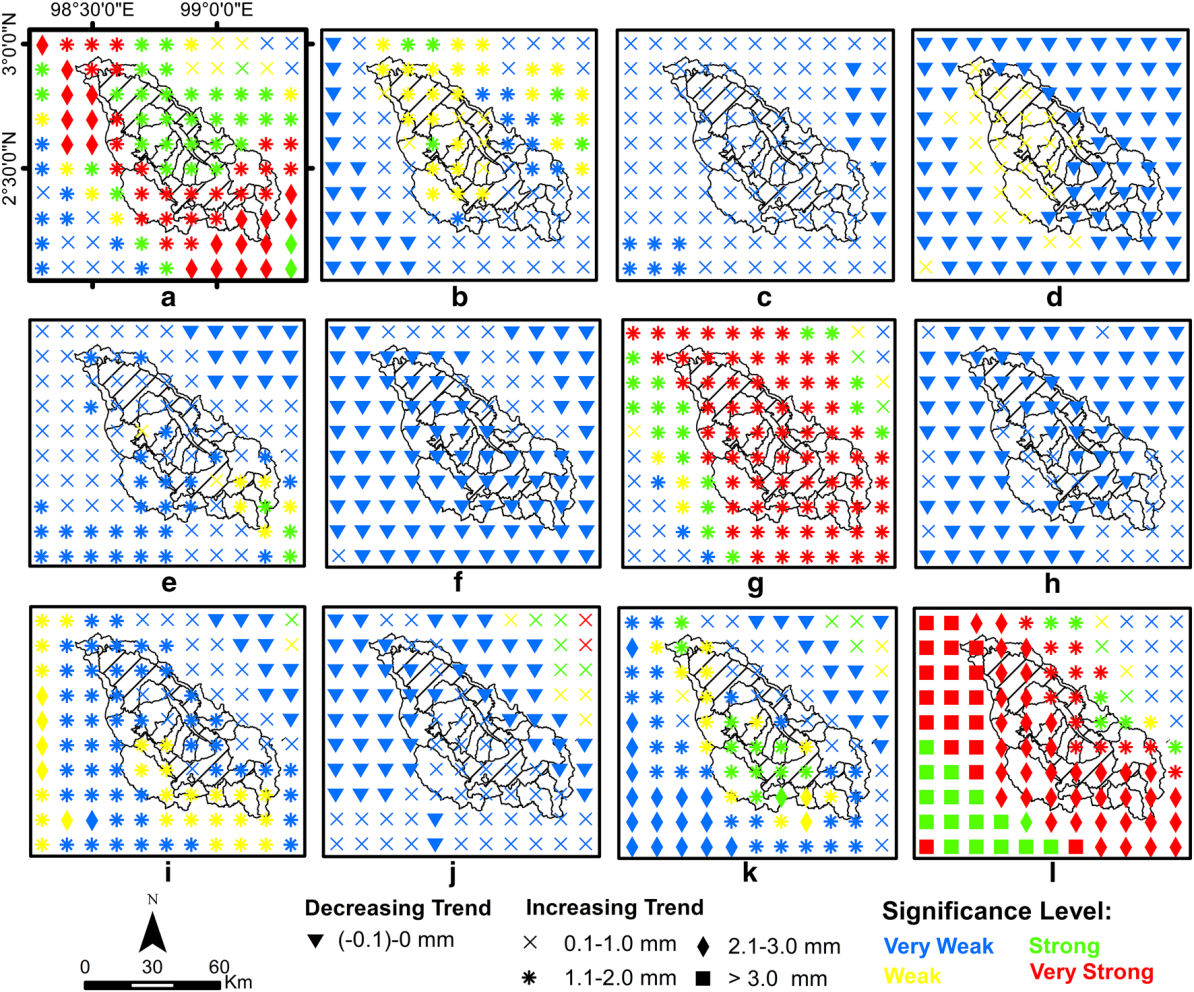
Spatial distribution of rainfall trends in the Lake Toba area for 1981–2020, (**a**) January, (**b**) February, (**c**) March, (**d**) April, (**e**) May, (**f**) June, (**g**) July, (**h**) August, (**i**) September, (**j**) October, (**k**) November and (**l**). December.

The spatial distribution of monthly rainfall trends in the study area, shown in Fig. 6, generally shows an increasing trend. The trends of increasing rainfall are very weak, weak, strong and very strong, at 39%, 8%, 8% and 15%, respectively, while the decreasing trend is very weak at 30%. The trend of increasing rainfall is distributed in January, February, April, June, August, October, November and December, while the decreasing trend of rainfall is distributed in March, May, July and September. The rates of increase in rainfall of 0.1–1 mm, 1.1–2 mm, 2.1–3 mm and > 3 mm per year are 31%, 30%, 7% and 3%, respectively. Meanwhile, the rate of decrease in rainfall of (-1)-0 mm per year is 30%, and the average increase rate is 0.71 mm per year. Generally, the trend of increasing rainfall occurs in the dry period and the second rainy season.

**Figure 7:**
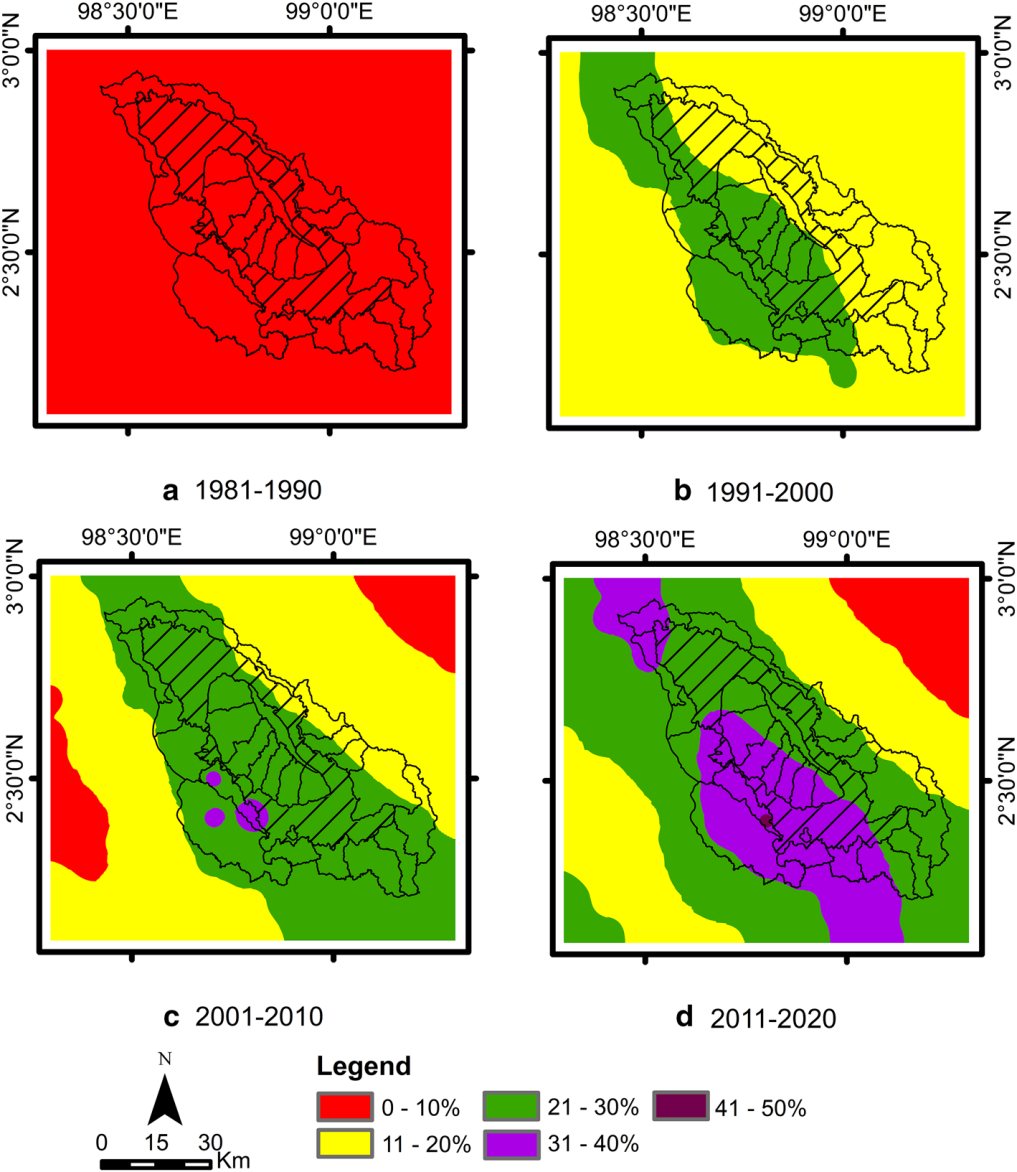
Spatial distribution of the percentage change in rainfall (**a**) 1981–1990, (**b**) 1991–2000, (**c**) 2001–2010 and (**d**) 2011–2020 relative to the period of 1951–1980.

The change in rainfall over a decade, as shown in Fig. 4, indicates a change in rainfall in the Lake Toba area. There was a change in rainfall around the study area, with the increasing trends for each decade of 6%, 17%, 17% and 22%, respectively. The spatial distribution shown in Fig. 7 also shows the same tendency, that is, an increase in rainfall of 0–10% in the early decades and an increase of 11–30% in the second and third decades. Generally, an increase of 21–30% is distributed in the western part of the Lake Toba catchment area in the second decade, while its distribution extends to the east and along the Bukit Barisan Mountains, stretching from north to south in the third decade. The fourth decade has the highest increase in rainfall, where the spatial distribution of the increase in rainfall of 21–40% is distributed in the Lake Toba catchment area and along the Bukit Barisan Mountains.

## Discussion

In all study areas, temperature shows a strong to very strong increasing trend in all seasons, except in the dry season period, and both trends are weak. The rate of increase in the last four decades has an average of 0.006 °C per year. This is relevant to an increase in the global average temperature of 0.006 °C per year from 1850 to 2020^1^. This also happens in several lakes around the world, such as in Lake Superior in North America^53^, Lake Urmia in Iran^54^, Lake Chad in Africa^55^ and Lake Dianchi in China^56^. The last decade (2011–2020) was the warmest decade, with an average increase of 0.24 °C relative to the base period. This is consistent with the study on Lake Chad^55^ and the trend of increasing global temperature^1^. A very strong trend of increasing temperature (α < 0.05) indicates that the region is vulnerable to climate change^57^.

In general, the trend of rainfall also shows an increasing trend. The increasing trend occurs in the dry season (except in July) and in the second rainy season (except for September). The decreasing trend occurs in the first rainy season. This indicates that the dry and rainy seasons are getting wetter with increasing rainfall. The increase in the dominant rainfall areas is 0.1–1 mm per year. The trend of increasing rainfall in the Lake Toba area is not in line with several conditions in other parts of the world that tend to have a decreasing trend of rainfall, as in Lake Superior in North America^53^, Lake Urmia in Iran^54^, Lake Chad in Africa^55^ and Lake Dianchi in China^56^. The period of 1981–1990 was the lowest decade of increasing rainfall, indicating a strong influence of the El Nino activity, which led to the decreasing rainfall^49^. According to The Intergovernmental Panel on Climate Change (IPCC) report on the increase of global average temperature^1^ a significant increase in rainfall in the Lake Toba area occurred in the last two decades.

The correlation between temperature and rainfall shows a linear trend where an increase in temperature affects an increase in rainfall. A significant increase in rainfall occurred in the catchment area of Lake Toba. This is due to the topography of the study area, since the Lake Toba area is surrounded by the Bukit Barisan Mountains, which stretch from the north to the south of Sumatra Island, and the existence of Samosir Island in the middle of the lake. An increase in temperature triggers an increase in evaporation in the Lake Toba area. At the same time, the mountains serve as a barrier so that the formation of rain clouds tends to occur in the water catchment area of Lake Toba. The local impacts caused by this condition need to be anticipated further to reduce the effects of climate change on extreme temperatures and rainfall, which might cause increasing hydrometeorological disasters. The occurrence of extreme temperatures has a bearing on forest and land fires as well as the potential for extreme rainfall that leads to floods and landslides in the Lake Toba area. While increasing rainfall, in general, can have beneficial impacts on agriculture and biodiversity, it can also lead to adverse socioeconomic problems, such as flooding. An increase in heavy or extreme rain also triggers more sediment flow into the lake^56^ and may potentially lead to silting in Lake Toba. Previous studies have shown variations in the water levels of Lake Toba that are also influenced by climate change and variability^13^.

## Conclusions

This study provides a clear picture of spatial and temporal variability as the impact of global climate change on temperature and rainfall trends over the last 40 years in the Lake Toba region of Indonesia. Global climate change causes increases and changes in temperature and rainfall in the Lake Toba region. In all study areas, there is a trend of increasing temperature, where the trend of increasing temperature is strong to very strong at 67% and weak at 33%. The average temperature increase is 0.006 °C per year. For rainfall, generally, there is a trend of increasing rainfall by 70%, while 30% indicates a decreasing trend of rainfall. The increase in rainfall is strong to very strong at 23%, and the increase is weak to very weak at 8% and 39%, respectively. The rate of increase in average rainfall is 0.71 mm per year. Generally, the trend of increasing rainfall occurs in the dry season and the second rainy season. Changes in temperature per decade relative to the base period indicate an increase in temperature at the study site for each decade; 0.09 °C, 0.06 °C, 0.15 °C and 0.24 °C, respectively. Meanwhile, rainfall shows the same pattern, namely, an increase in rainfall for each decade of 6%, 17%, 17% and 22%, respectively. The last decade was the hottest decade, with a significant increase in temperature ranging from 0.21 to 0.3 °C, but in some parts of the Lake Toba catchment area, the temperature increase was 0.11–0.2 °C lower. This study also explains that the ERA5-Land data have been well corrected by the QM method using a quantile value of 4 for temperature and 80 for rainfall. This study is expected to be a valuable input for the government, society and policy makers in implementing climate change adaptation and mitigation programs in the Lake Toba region for the sustainable use of lake water resources.

## Data Availability

The temperature and rainfall data for 1981–2020 used in this study were taken from the BMKG. ERA5-Land data downloaded from (https://cds.climate.copernicus.eu). The software used in the analysis is Matlab R2022a (License ID: 41112927, Student-Individual) and ArcGIS Desktop 10.8 (License ID: ESU057739221). While the base map of the Lake Toba water catchment area is sourced from the Asahan Barumun Watershed and Protected Forest Management Agency (BPDASHL), the base map of Sumatra Island is sourced from the Geospatial Information Agency, and the DEM SRTM 30 m topographic map is sourced from https://drive.google.com/file/d/1nZ1ed9j2wWexh4SQ-HuFVhIYR88Rfon0/view.
